# The use of thermographic imaging to evaluate therapeutic response in human tumour xenograft models

**DOI:** 10.1038/srep31136

**Published:** 2016-08-05

**Authors:** Nosheen Hussain, David Connah, Hassan Ugail, Patricia A. Cooper, Robert A. Falconer, Laurence H. Patterson, Steven D. Shnyder

**Affiliations:** 1 University of Bradford, Institute of Cancer Therapeutics, Bradford BD7 1DP, United Kingdom; 2 University of Bradford, Centre for Visual Computing, Bradford BD7 1DP, United Kingdom

## Abstract

Non-invasive methods to monitor tumour growth are an important goal in cancer drug development. Thermographic imaging systems offer potential in this area, since a change in temperature is known to be induced due to changes within the tumour microenvironment. This study demonstrates that this imaging modality can be applied to a broad range of tumour xenografts and also, for the first time, the methodology’s suitability to assess anti-cancer agent efficacy. Mice bearing subcutaneously implanted H460 lung cancer xenografts were treated with a novel vascular disrupting agent, ICT-2552, and the cytotoxin doxorubicin. The effects on tumour temperature were assessed using thermographic imaging over the first 6 hours post-administration and subsequently a further 7 days. For ICT-2552 a significant initial temperature drop was observed, whilst for both agents a significant temperature drop was seen compared to controls over the longer time period. Thus thermographic imaging can detect functional differences (manifesting as temperature reductions) in the tumour response to these anti-cancer agents compared to controls. Importantly, these effects can be detected in the first few hours following treatment and therefore the tumour is observable non-invasively. As discussed, this technique will have considerable 3Rs benefits in terms of reduction and refinement of animal use.

Human subcutaneous (s.c.) tumour xenografts play a key role in the pre-clinical development of novel anti-cancer agents. Human tumour cells or tumour fragments are subcutaneously transplanted into the flanks of immunodeficient mice, and once tumour is detectable visually or by palpation, drug treatment commences, with tumour growth measured using callipers to determine tumour cross sectional area as a function of tumour volume. Tumour growth delay following treatment is compared with untreated controls to determine any drug effects. These models often suffer from low accuracy and poor reproducibility^1^, with the additional problem that the use of callipers causes mechanical disruption to the tumour leading to unreliable growth monitoring. Furthermore, when evaluating agents which provoke a rapid effect, there are issues with detection of tumour volume change^2^. In order to assess such activity, the mouse must be sacrificed and the tumour assessed *ex vivo* for any changes. As a result, the effect is not tracked in the same mouse over time and a large number of mice are required. There is therefore a need for techniques which enable evaluation of drug efficacy, particularly in the first few hours after administration, to circumvent the issues raised.

One powerful solution to this problem is to employ non-invasive imaging techniques to monitor tumour growth and vasculature changes. To this end, a wide variety of methods have been explored. Techniques such as MRI and ultrasound are known to be effective in estimating tumour size and drug-tumour interactions^3^, but they require highly specialised and expensive equipment which restrict their application, and usually for animals, there is also a requirement that the animals be restrained using anaesthesia while scanning. There is thus a need for inexpensive, portable methodologies which can be used on lightly restrained, non-anaesthetised animals, and non-invasive infrared thermography (also known as thermographic imaging) is such a technique.

Thermographic imaging can monitor both tumour growth and vasculature changes in s.c. implanted xenografts. Infrared thermography measures infrared radiation in the 7,000–15,000 nm range, which is then related to the temperature of the object being imaged^4^. This method can be used to take a temperature reading from the surface of the skin. The skin is known to be an important thermoregulator, whereby heat is transferred to and from the skin via three mechanisms: conduction through tissue layers, convection through the vasculature, and radiation^5^. As such, we rationalised that measurement of skin temperature may be a way to indirectly measure the vasculature of a particular underlying tumour region, and would therefore be an effective way of monitoring therapy effects.

Thermographic imaging has already been applied to the detection of tumours, as a means to aid diagnosis of clinical breast cancer, where due to inflammation local to the tumour, it is warmer than the surrounding tissue^6^. Thermographic imaging has also been applied to breast tumour xenografts in small animals^7^,^8^,^9^, where in this case the tumour is cooler than its surroundings, which the authors suggested may be due to the use of immunocompromised mice and the lack of an inflammatory response^7^. Furthermore, an increase in tumour size has been associated with a decrease in tumour temperature in comparison with the surrounding tissue, suggesting that thermographic imaging might be an indirect means of monitoring tumour size^8^.

In this study we directly assess the potential for thermographic imaging to be applied to the evaluation of novel therapeutics in the s.c. tumour xenograft model. Firstly, the detection of xenografts of different tissue origins is studied to confirm that the technique can be applied to tumour types beyond breast cancer. Secondly the suitability of the methodology to assess the efficacy of anti-tumour agents with distinctly different mechanisms of action is evaluated.

## Results

### Thermographic imaging clearly delineates the area of the tumour xenograft, with imaging possible for a range of tumour types

**Figure 1:**
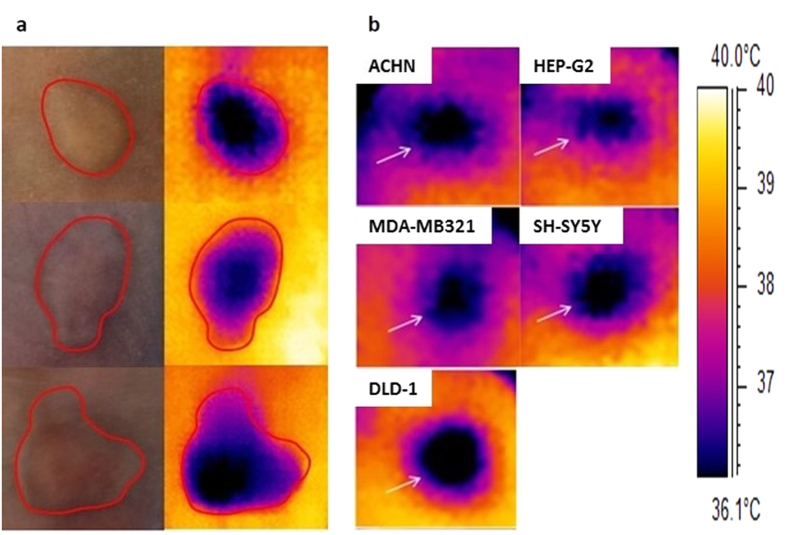
Thermographic imaging clearly delineates the area of the tumour xenograft, with imaging possible for a range of tumour types. **(a**) Sample images of H460 NSCLC tumour xenografts captured using a thermal camera (right hand side) alongside images captured with a standard digital camera (left hand side). Darker, bluer colours denote cooler temperatures. The area of tumour is marked out in digital images (red lines) on both sides, and shows that there is a clear reduction in temperature at the tumour location. (**b**) Thermal images of tumours for xenografts covering a range of original tissue types (tumour boundary identified by white arrows); tumour types include ACHN (renal), DLD-1 (colon), HEP-G2 (liver), MDA-MB-21 (mammary), SH-SY5Y (neuroblastoma).

To demonstrate the extent to which tumours can be discriminated based on the temperature differential with the surrounding tissue, corresponding thermal and digital images were taken of the same H460 tumour xenograft. It can be seen that the area of significant temperature drop was contained within the borders of the tumour area as marked out on digital images taken of the same tumour xenograft (Fig. 1a). In addition, five other tumour xenografts covering a range of different origin tissue types were imaged. In all cases a clear decrease in tumour temperature was seen when compared to the mouse flank temperature (Fig. 1b).

### Treatment with ICT-2552 results in a significant initial temperature drop, whilst a significant drop in tumour temperature relative to the tumour volume is observed over the longer time period for both compounds

**Figure 2:**
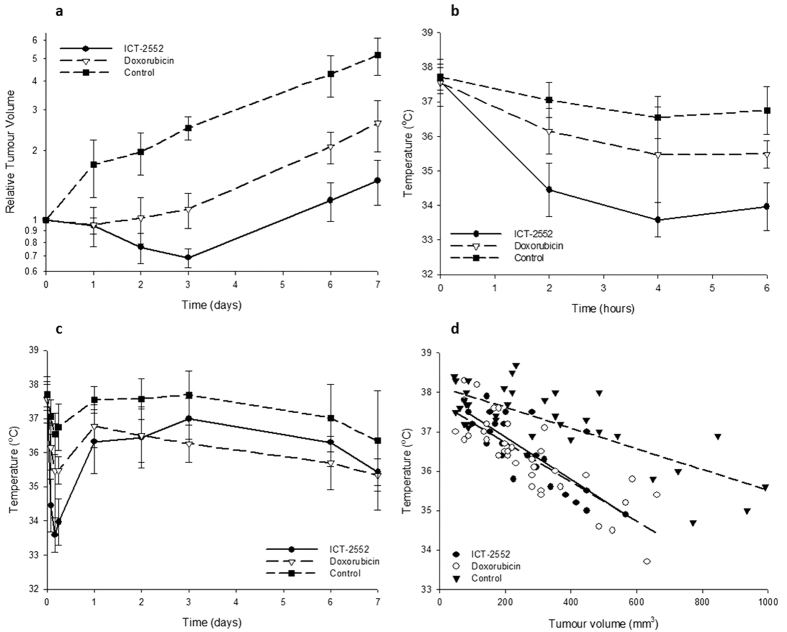
Treatment with ICT-2552 results in a significant initial temperature drop, whilst use of either therapy leads to a significant drop in tumour temperature relative to the tumour volume over the longer time period. (**a**) Evaluation of the efficacy of ICT-2552 and doxorubicin in terms of relative tumour volume over time (volume as a multiplicative factor of volume at day 0) based on calliper measurements. Results show a significant reduction in tumour volume for the 2 treatments relative to the control. (**b**) Plot of temperature versus time for all three treatment groups over the first 6 hours; curves show the mean of the tumour temperatures within the group. There is a temperature reduction for all three groups, but this is significantly greater for ICT-2552 than doxorubicin (p = 0.006) and the controls (p < 0.001). (**c**) plot of time versus temperature for daily time points; curve shows mean temperature for tumours within each group. A recovery is seen after 24 hours and then a gradual decrease in temperature as the tumour grows across all groups (p < 0.01 for all groups). (**d**) Scatter plot of temperature versus tumour volume for the three treatment groups, with lines of best fit shown. While tumour size is proportional to tumour temperature for all groups, we found that the slope of the line-of-best-fit was much steeper for the treated groups (p < 0.001).

Both agents demonstrated efficacy (p < 0.05 for ICT-2552, and p < 0.05 for doxorubicin) in the H460 tumour model using standard calliper measurement (Fig. 2a). Assessing tumour temperature (Fig. 2b), over the first 6 hours following treatment, the temperature drop observed for the control group was small (<1 °C compared to the initial reading) but significant (p < 0.001). For the doxorubicin group the temperature drop was slightly larger (>2 °C) and was significantly different from the control (p = 0.004), whilst the ICT-2552 group had the greatest drop in temperature (approximately 4 °C) which was significantly lower than both the control (p < 0.001) and doxorubicin treated groups (p = 0.006). As would be expected with a single treatment of ICT-2552, the vasculature recovers after 24 hours accompanied by a rise temperature, and with doxorubicin there was also recovery after 24 hours.

Between 24 hours and 7 days an overall drop in temperature for all three groups was seen (Fig. 2c). Linear regression analysis shows that this trend is highly significant for each group, and the magnitude of that drop is also similar for each group: for controls the change in temperatures was −0.173 °C/day (p = 0.003); for doxorubicin −0.274 °C/day (p < 0.001), and for ICT-2552 −0.199 °C/day (p = 0.005).

When the relationship between tumour temperature and absolute tumour volume was investigated, a linear relationship between the 2 parameters was observed (Fig. 2d). To quantify this relationship, a linear model was applied to each individual treatment group, and to all groups together. Each individual group showed a significant negative relationship between volume and temperature (i.e. increases in volume are correlated with decreases in temperature): the control group showed a reduction of 0.0026 °C/mm^3^ (p < 0.001), doxorubicin showed a reduction of 0.0050 °C/mm^3^ (p < 0.001) and ICT-2552 showed a reduction of 0.0054 °C/mm^3^ (p < 0.001). Interestingly, treatment groups showed a larger drop in temperature with increase in volume compared to the control group, suggesting that therapeutic intervention has remodelled the tumour with an associated reduction in vascularisation with a consequence of reduced temperature/tumour volume.

### Bodyweight loss seen for doxorubicin treatment is mirrored by reduction in body temperature

**Figure 3:**
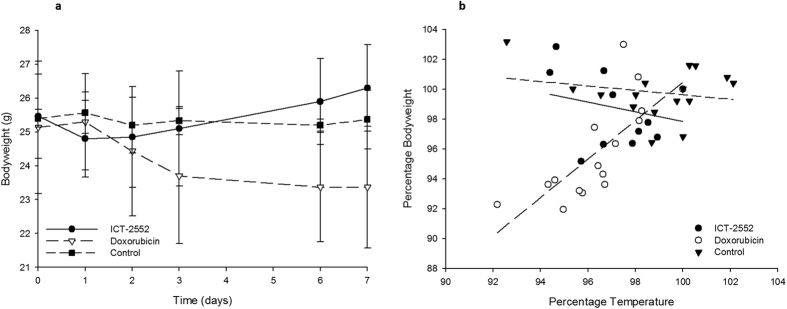
Weight loss seen for doxorubicin treatment is mirrored by reduction in body temperature. **(a**) bodyweight curve showing weight loss as a function of time. Doxorubicin shows a reduced weight relative to the other groups over time (p < 0.01). (**b**) a scatter plot showing weight loss versus temperature for each group, with the line of best fit superimposed over the data. A linear relationship was seen between the two parameters for the cytotoxic doxorubicin, but not for ICT-2552 or the control group.

Significantly more bodyweight loss was observed over the course of the experiment for mice treated with doxorubicin, compared to those treated with ICT-2552 (p < 0.01) or untreated controls (p < 0.001), with no difference between ICT-2552 and controls (p = 0.61) (Fig. 3a). When body temperature was measured and the relationship with weight loss investigated (Fig. 3b), a linear relationship was observed for doxorubicin but not for the other two groups, suggesting that the toxic effects of doxorubicin can be detected by thermography.

## Discussion

In this study the application of the thermographic imaging technique to detection of xenografts of different tumour types has been demonstrated, expanding on previously published studies which focused solely on growth of breast cancer xenografts^7^,^8^. For the first time we demonstrate the utility of thermographic imaging for measurement of the response of tumour xenografts to anti-cancer chemotherapies.

The main finding of the work presented here is that thermographic imaging can be used to non-invasively monitor the therapeutic and toxic effects of administering different classes of anti-cancer agents to mice bearing s.c. tumour xenografts. Crucially we have shown that this monitoring is possible in the first 6 hours after treatment, a period in which the tumour is not sufficiently grown to measure using conventional methods, e.g. callipers. This will bring significant 3Rs benefits, since monitoring can be carried out in the same animal over the entire length of the experiment rather than having to use cohorts of animals for each time point^10^.

Specifically, we have successfully demonstrated that thermographic imaging can be readily utilised to monitor the early onset effects of a vascular disrupting agent (VDA), the experimental compound ICT-2552. In previous studies it has been demonstrated that vascular shutdown occurs within the first 6 hours of compound administration for this^11^ and other VDAs^12^,^13^, with subsequent re-opening/recovery of the vasculature after 24 hours. In this study, a significant temperature reduction was observed at 6 hours with subsequent increase. This is consistent with a closure of the vasculature in the tumour leading to a reduction in blood flow concomitant with a reduction in temperature. Whilst an increase in temperature is then seen over the next 24 hours, as the vasculature is re-opened^14^, there is then a subsequent drop at a higher rate in relation to the control as the tumour grows. This could be explained by drug treatment leading to progressive tumour necrosis with an associated loss of tumour vasculature concomitant with the progressive observed temperature decrease.

With doxorubicin, which has a different mechanism of action to ICT-2552^15^, an increased rate of tumour temperature reduction compared to the untreated control was observed to a lesser extent. In contrast to the direct effect on tumour vasculature leading to temperature reduction, with doxorubicin it is likely to be due to increased tumour necrosis indirectly leading to a reduced vasculature and subsequently reduced temperature^16^.

Although this study was not specifically focused on the application of thermographic imaging to monitor off-target drug toxicities, the relationship between body weight loss and temperature drop would suggest that thermographic imaging could also be utilised to monitor overt treatment toxicity.

Whilst other non-invasive technologies are available to monitor therapeutic effects in cancer, such as the use of ultrasound, positron emission tomography or magnetic resonance imaging^3^, thermographic imaging clearly has many advantages, in particular its ease of use and portability. Thermal cameras are also cost effective and readily available. The equipment and associated software for analysis requires limited training and has a variety of applications from diagnosis through to response, treatment and prognosis. As well as eliminating the need to use invasive procedures, thermal radiation is passive, thus no animal would be exposed to harmful radiation giving the procedure significant 3Rs benefits. Using thermographic imaging enables the tracking of the drug in a single mouse over the study duration, thus reducing the number of animals required for experimentation. In addition thermography could replace mechanical callipers to check tumour volume thus refining the process as the handling of the animal and calliper pressure placed on the tumour is minimised.

In conclusion, we have demonstrated that thermographic imaging can be used to assess tumour growth and therapeutic intervention non-invasively in a broad range of tumour types in preclinical cancer models. Whilst further research is required to fully understand the biology underpinning the concept behind therapeutic agent-associated temperature reduction, and on translating the technology for clinical use, thermographic imaging is shown to be a rapid, reliable and cost-effective technology for use in preclinical studies of anticancer agents.

## Methods

### Imaging equipment

An FLIR ThermoVision™ A-Series Infrared Camera A320 (FLIR Systems AB, Danderyd, Sweden) was used. This uses a focal plane array sensor with uncooled microbolometer and has a spectral range of 7.5–13 μm. The captured image has a size of 320 × 240 pixels, at 16 bits per pixel, and a temporal frequency of 9 Hz. The imaging system has a fixed aperture of 1.3 F stops, a focal length of 18 mm and a minimum focus distance of 40 cm. All measurements were taken in a temperature-controlled room. The camera was calibrated by imaging a 37 °C-preheated squash ball at the beginning and end of every session.

### Compounds

The experimental vascular disrupting agent (VDA), azademethylcolchicine (ICT-2552) was synthesized in-house as described previously^11^ and was administered in 10% DMSO: 90% arachis oil. Doxorubicin (Sigma, Poole, UK) was administered in saline. Compounds were administered at or below their maximum tolerated dose.

### Tumour xenografts

Balb/c immunodeficient nude mice (Envigo, Loughborough, U.K.), between the ages of 6 and 8 weeks were used. Throughout the study, all mice were housed in air-conditioned rooms in facilities approved by the United Kingdom Home Office to meet all current regulations and standards. All procedures were carried out under a Project Licence (PPL 40/3670) issued by the UK Home Office according to government legislation, following approval of the work by the local Animal Welfare Ethics Review Board at the University of Bradford, and in accordance with the UK National Cancer Research Institute Guidelines for the Welfare of Animals^17^. Tumour xenografts were established as previously described in the left and right flanks under brief general inhalation anaesthesia^18^.

### Evaluation of therapy

The H460 lung adenocarcinoma model was selected for these studies. Once tumour volumes reached approximately 32 mm^3^ (as measured by callipers) mice were randomised into groups (n = 3) and received either ICT-2552 at 20 mgkg^−1^, or doxorubicin at 10 mgkg^−1^, as a single intraperitoneal dose on day 0. For comparison, a control group was left untreated since extensive previous studies in-house using the excipients in this study have not demonstrated any effect on tumour growth, and therefore it was not deemed ethical to set up excipient only control groups in addition to the untreated control. Tumour volume was determined using callipers as follows: 2-dimensional calliper measurements of the tumours were taken, with volumes calculated using the formula (*a*^2^ × *b*)/2, where *a* is the smaller and *b* the larger diameter of the tumour. Tumour volume was then normalised to the respective volume on day 0, and semi-log plots of relative tumour volume (RTV) versus time were made. Animal body weights were recorded throughout the experiment to monitor for any deleterious effects. Mann-Whitney U tests were conducted to determine the statistical significance of any differences in growth rate (based on tumour volume doubling time) between control and treated groups. In addition thermographic images were captured as described below.

### Thermographic imaging of xenografts

To capture thermographic images, non-anaesthetised mice were held at a fixed distance of 50 cm from the camera and a sequence of images captured over a 3 second period, generating approximately 30 images. Individual images were chosen from the sequence using visual assessment, with the images demonstrating the sharpest edges selected to reduce the effect of motion blur caused by mouse movement. Immediately following thermographic imaging, digital images were captured using a Panasonic Lumix SZ1 digital camera used in macro mode with flash disabled. The thermal images were analysed with ThermaCam Researcher Pro 2.10 software (FLIR Systems AB, Danderyd, Sweden). The mean, minimum and maximum temperatures across each tumour were calculated by drawing a straight line along the maximum tumour diameter and capturing temperature data across the line.

### Statistics

Two-tailed t-tests were carried out at 95% confidence interval, including Bonferroni correction for multiple comparisons, and, to compare the relationship between tumour temperature and both body weight and tumour volume, a linear regression to fit a linear model to the data was used, with an F-test used to assess accuracy.

## Additional Information

**How to cite this article**: Hussain, N. *et al.* The use of thermographic imaging to evaluate therapeutic response in human tumour xenograft models. *Sci. Rep.* **6**, 31136; doi: 10.1038/srep31136 (2016).
